# A comparison of available guidelines for the detection of cauda equina syndrome and assessing the need for further clinical guidance in Ireland

**DOI:** 10.1007/s11845-024-03633-5

**Published:** 2024-03-20

**Authors:** Lorcan Gavin, Michael G. Curran, John P. McCabe

**Affiliations:** 1 University of Galway, University Road, Co Galway, Ireland; 2 Department of Trauma and Orthopaedics, Bon Secours Hospital, Renmore, Galway, Ireland

**Keywords:** Cauda equina syndrome, CES, Detection, Guideline, Investigation

## Abstract

The cauda equina syndrome (CES) is a rare but critical disorder, which can result in devastating motor weakness and sensory deficit, alongside often irreversible bladder, bowel and sexual dysfunction. In addition to the clinical burden of disease, this syndrome results in a disproportionately high medicolegal strain due to missed or delayed diagnoses. Despite being an emergency diagnosis, often necessitating urgent surgical decompression to treat, we believe there is a lack of clarity for clinicians in the current literature, with no published Irish guideline concerning screening or detection. The current study aims to identify and analyse appropriate guidelines in relation to CES screening which are available to clinicians in Ireland. The study design included a comprehensive literature review and comparison of existing guidelines. The review identified 13 sources of appropriate guidance for clinicians working in Ireland. These resources included textbooks, websites and guidelines developed in the UK. No Irish guidelines or advice were available on CES screening/treatment at the time of review. This review demonstrated the lack of consensus and guidance for clinicians in Ireland on how to effectively screen for CES, judge who requires further imaging and investigations and how to rule out the condition. A national consensus on thorough screening and prompt investigation for CES is necessary, and the formulation of new CES guidelines would be a welcome addition to what is available to clinicians currently.

## Introduction

The cauda equina syndrome (CES) is an uncommon but potentially debilitating condition, resulting from compression or damage to the lumbosacral nerve roots of the cauda equina. This is a disabling condition that, if undetected and untreated, can result in severe irreversible disability. Anatomically, the cauda equina is a collection of nerve roots that emerge from the spinal cord at its termination in the lumbar spine, which innervates the lower limbs, perineum and pelvic organs. Dysfunction in these nerve roots as they exit their respective neural foramina results in the clinical manifestations of CES.

The aetiology of CES incorporates a range of causative factors, including disc herniation [1, 2], spinal stenosis [3], trauma [4–6] and post-surgical cases [6, 7]. Ultimately, any mass or compressive effect in the lumbosacral canal that could result in nerve root dysfunction should be considered as a cause, and cases have also been attributed to infective [8, 9], inflammatory [10] and vascular [11, 12] disorders, respectively. Consequently, it is important to consider both primary tumours and secondary metastatic disease as causative pathologies in CES diagnoses, especially given the reported rising rate of metastatic bone disease in Ireland [13].

A broad range of symptoms is attributed to CES in the literature including severe lower back pain, sciatica which refers to radiating pain into the lower limbs, paresthesia and sensory loss (especially perineal or perianal sensory loss or saddle anaesthesia), weakness and loss of reflexes alongside urinary, faecal and sexual dysfunction. The three classical cardinal symptoms, of which at least one is historically required for a diagnosis to be made as per Fraser et al. [14], are bladder or bowel dysfunction, reduced sensation in the saddle area and sexual dysfunction with possible neurological deficit in the lower limb.

Fortunately, CES is a rare disorder, with crude incidence rates varying between 0.3 and 7 per 100,000, depending on the surveyed geographical location [15–17]. However, due to the comprehensive network of innervations distal to a potential CES lesion, the implications of disease can be catastrophic. Long-term impairment can leave patients unable to self-care and reliant on mobility aids, urinary catheterisation and bowel regimes.

Following the onset of disease, the speed at which treatment, usually consisting of surgical decompression of the compressed nerve roots, is commenced is critical and is proven to be of huge importance to successful patient outcomes and the avoidance of permanent deficits [2, 18]. Early diagnoses and treatment have been seen to result in satisfactory patient outcomes [17], whilst if untreated due to delayed diagnosis, CES may result in severe irreversible disability [18, 19]. In fact, even in patients who present with severe deficits, high rates of improvement can be seen with prompt intervention [20–22]. Diagnosis of CES requires clinical and radiological correlation, most often by referral for magnetic resonance imaging (MRI) of the spine. Clinical history, exam and imaging studies are each in isolation not enough to confirm diagnosis but should be carefully considered together [23, 24]. The rarity of disease, coupled with the subtlety and variety of symptoms, and compounded by inconsistent provision of MRI scanning, can unfortunately lead to CES being missed in a triage setting.

These avoidable delays and irreversible fallouts result in a large patient burden, at a huge medicolegal and financial cost [25]. Thus, we proposed a review of the available literature to locate, compare and provide an overview of the available guidelines for the investigation and detection of CES. By outlining their key recommendations and the challenges associated, we aim to improve clinicians understanding of this complex condition, whilst also highlighting the need for, and hopefully inspiring the implementation of, a similar structured clinical guidance in Ireland.

## Methods and materials

A comprehensive search was conducted to assess what resources were available to guide a clinician in Ireland when faced with a potential case of cauda equina syndrome. This included searches of Scopus and PubMed using combinations of keywords: cauda equina syndrome, guideline, investigation, detection, and diagnosis. A range of literature concerning CES was reviewed to add context to the studied guidelines.

In addition, searches were carried out of the websites of the Health Service Executive of Ireland (HSE), the UK’s National Health Service (NHS), the National Institute for Health and Care Excellence (NICE), the British Association of Spinal Surgeons (BASS), the Society of British Neurological Surgeons (SBNS), the British Medical Journal (BMJ), the American Association of Neurological Surgeons (AANS) and the American College of Radiology (ACR) to ascertain what guidance these organisations provided. Two orthopaedic textbooks, an orthopaedic educational website and two textbooks for general practice physicians were also reviewed for guidance.

Chosen sources were limited to papers and guidelines that were published in the English language and that were deemed reasonably available to physicians in Ireland. No time delimiters were placed on the date of publications, but where available, more recently updated or reviewed guidelines were preferred. Where percentages are used to compare sources, they are rounded to the nearest whole per cent. In total, thirteen sources met the inclusion criteria for this study and were reviewed. Advice from various other cited studies is also referenced, as is the relevant literature regarding cauda equina syndrome. No new ethical approval was needed for this project. No new patient data was utilised, and therefore, new ethical approval was not needed.

## Results

Thirteen distinct guidelines or sources providing advice on the screening for CES were reviewed for this paper and are included in Table 1.

**Table 1:** Final list of resources included for review

	**Name**	**Type of source**	**Country of origin or publication**
1	NICE guidelines and clinical knowledge summary (CKS)	Guideline	UK
2	NHS GIRFT ‘Get It Right First Time’ guideline	Guideline	UK
3	British Association of Spine Surgeons/Society of British Neurological Surgeons (BASS/SBNS) guideline	Guideline	UK
4	*British Medical Journal* (BMJ) best practise guideline	Guideline	UK
5	American College of Radiology (ACR) imaging guideline	Guideline	United States of America
6	American Association of Neurological Surgeons (AANS) advice	Guideline	United States of America
7	NICE guidelines and clinical knowledge summary (CKS)	Guideline	UK
8	Up to date guidelines	Website	Netherlands/international
9	Orthobullets, orthopaedics training website	Website	United States of America
10	The textbook of spinal surgery (Bridwell and DeWald)	Book	United States of America
11	The Adult and Pediatric Spine (Frymoyer, Lauerman)	Book	United States of America
12	Oxford handbook of clinical medicine	Book	UK
13	Practical general practise (GP textbook)	Book	UK

These included NICE guidelines and clinical knowledge summaries (CKS) [26], the Oxford Handbook of Clinical Medicine [27], a university recommended GP textbook [28], two spinal orthopaedic textbooks [29, 30], guidelines from UpToDate’s website [31, 32], the BASS/SBNS guidelines [33], the BMJ Best Practise guidelines [34], the NHS ‘Get It Right First Time’ (GIRFT) pathway [35], an NHS local guideline [36], ACR image appropriateness guidelines [37], patient information from the AANS website [38] and the orthopaedic educational website ‘Orthobullets’ [39]. Whilst not exhaustive, these sources were deemed comprehensive in scope and broadly representative of what is currently available to clinicians.

The format observed in each of the thirteen guidelines was fundamentally quite similar, with each source advising clinicians to observe patients for several key symptoms, often referred to as ‘red flags’, and if these were present to then investigate accordingly. The symptoms deemed to require investigation differed between sources, as delineated by in the table (Table 2).

**Table 2:** Frequencies of the ‘red flag’ symptoms (underlined) and specific sub-symptoms amongst the sources assessed

**‘Red flags’**	**Frequency (%)**
Back pain	13 (100%)
- Leg pain	- 11 (85%)
- Sciatica	- 4 (30%)
- Radiculopathy	- 3 (23%)
Leg weakness	11 (85%)
- Bilateral	- 2 (15%)
Lower limb/perineal sensory deficit	13 (100%)
- Saddle anaesthesia	- 12 (92%)
- Bilateral (lower limb)	- 6 (46%)
Urinary dysfunction	13 (100%)
- Retention	- 4 (30%)
- Incontinence	- 2 (15%)
Bowel dysfunction	12 (92%)
- Laxity of anal sphincter	- 5 (38%)
- Rectal fullness	- 4 (30%)
Sexual dysfunction	6 (46%)

**Fig. 1:**
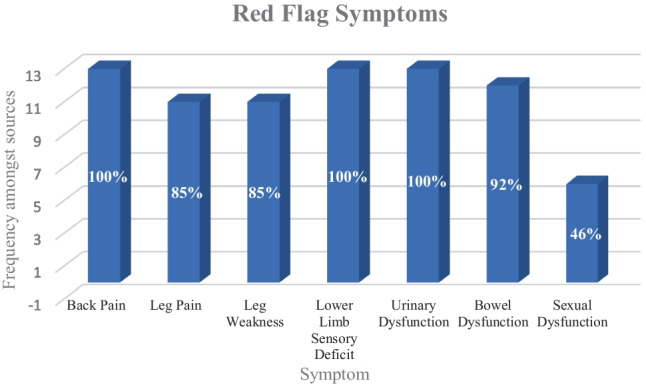
Frequency of documentation of ‘red flag’ symptoms within the sources assessed

All thirteen guidelines described back pain, some form of perineal or lower limb sensory deficit and urinary dysfunction (100%). Bowel and/or rectal dysfunction was described in 12 of the 13 guidelines (92%), with unilateral or bilateral leg pain described in 11 (85%). Leg weakness was also described in 11 of 13 (85%), with 2 of the sources specifying leg weakness to be bilateral (15%). Saddle anaesthesia was mentioned 12 times (92%), sciatica in 4 (30%) and radiculopathy in 3 (23%) (Fig. 1).

With regard to urinary function, retention and incontinence were specified in four (30%) and two cases (15%), respectively. Five of the sources specified laxity upon examination of the anal sphincter (38%) with another four specifying rectal fullness (30%). Six of the sources mentioned sexual dysfunction as a possible screening symptom (46%).

**Fig. 2:**
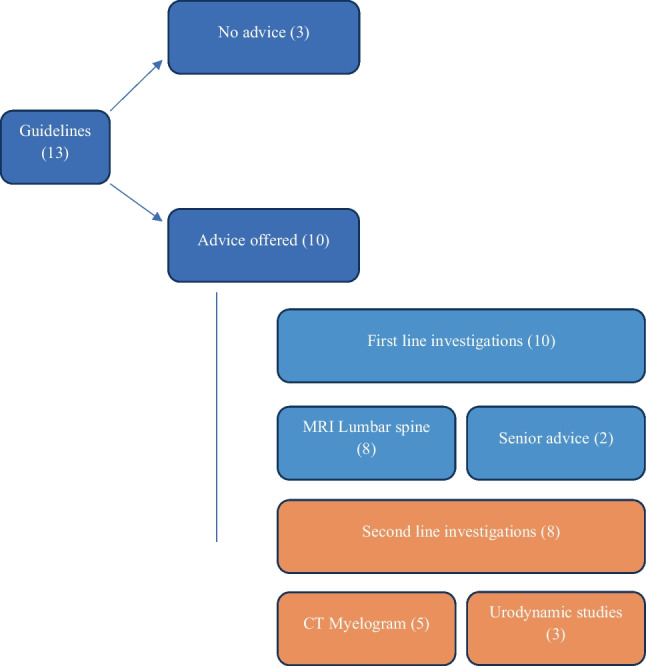
Summary of investigative advice provided by sources assessed

Overall, 10 of 13 sources reviewed (77%) offered advice on what immediate action to take to investigate possible CES. Amongst those that offered advice, there was a strong consensus on the first choice for investigative modality, with eight (80%) recommending urgent MRI of the lumbar spine. Five of the ten offered a second choice of computed tomography (CT) myelography scanning of the spine (50%), in the case that MRI spine was contraindicated or unavailable, whilst two advised clinicians to refer urgently to orthopaedic surgeons or neurosurgeons (20%) for specialist advice and a further three sources reviewed (30%) mentioned urodynamic studies or post-void bladder (PVB) scanning as a supplementary investigation in patients with suspected CES. Three of thirteen guidelines did not specifically advise in relation to imaging choice or further investigation (23%). A summary of advice is furnished in the figure (Fig. 2).

## Discussion

Through review of the available relevant literature, the authors feel we have replicated succinctly what guidance is available to clinicians who are faced with potential cases of CES. Notably, no guidance was readily available from any source in Ireland, including any HSE guidance. Similarly, no recommendation has been made in Ireland for the adoption of a specific international guideline, which creates a lack of clarity in relation to what guidance may prove beneficial. In addition, gaining access to the guidance that is available internationally is not altogether undemanding. Whilst the NICE CKS summaries are useful, straightforward tools, they are only readily available online for clinicians based in the UK, Crown Dependencies and British Overseas Territories. Similarly, the recent GIRFT interactive guideline [35] provides a comprehensive guide to screening for potential CES; however, some of the hyperlinks in this click-through pathway require subscription to British- or UK-based organisations such as the BASS. Other resources, such as the two textbooks reviewed [27, 28], offered valuable information, but their practicality in the screening for an acute diagnosis is questionable. Whilst there is valuable guidance available, the lack of clarity in choice and ease of access is undoubtedly a hindrance to Irish clinicians.

One possible aid to resolving any ambiguity around screening may be to clarify some of the difficulties around the definition of CES. Historically, the literature surrounding CES has been noted for its inconsistencies in definition, with multiple classifications proposed by different authors [40, 41]. Some publications, especially that of Fraser et al. [14] and Gleave and McFarlane [42], have attempted to clarify this, by establishing a multi-tiered approach to CES. This concept includes the dyadic definition of an initial incomplete CES (CESI), which may progress to CES with painless bladder retention (CESR). Gleave and McFarlane characterised the incomplete syndrome CESI as having ‘altered urinary sensation, loss of desire to void, poor urinary stream and the need to strain in order to micturate’ and distinguished this from the ‘painless urinary retention and overflow incontinence where the bladder is no longer under executive control’ of CESR [42]. Lavy et al.’s study expanded upon this, recommending the addition of the supplementary categories CESS, CESE and CESC for ‘suspected’, ‘early’ and ‘complete’ CES, respectively [43]. Whilst the definition of CES is not the focus of this paper, it is important to consider, as a categorical approach implies a spectrum of severity, prognosis and thus a variance in the haste at which intervention should be advised in potential guidance. If the universal adoption of a specific definition was to limit the ambiguity around definition, it would be valuable in the formulation of further guidance.

No symptoms or sign has been seen to have absolute predictive value for establishing a diagnosis for CES, and as such, most of the guidance assessed used clinical history and examination to screen for red flag symptoms. However, for a screening reliant on these two user techniques, little advice is offered to clinicians in terms of the content and structure of their history and exam. If we consider the AANS advice, for example [38], the ‘importance of clinical history and exam’ is stressed, but what exactly this exam should entail is not detailed.

The published literature [44, 45] questions the predictive value of clinical examinations such as a digital rectal exam [46], but exam findings such as reduced perianal tone are listed as ‘red flags’ for referral. It should be noted also that the specific examinations that are mentioned for use in assessing for CES, such as assessing for perianal sensation, disturbed saddle sensation or reduced anal tone, may not be examinations that many clinicians do often, and given the oft subtle nature of CES symptoms, guidance towards these would be welcomed on a screening tool. Whilst the evidence for the use of patient history and exam to diagnose CES outright is admittedly low [23], the triage interaction is the major determinant in decision for referral for MRI investigation and therefore should be a comprehensive guided process.

The ‘red flag’ approach is a near constant in the available guidance for screening for CES. Most guidance advises clinicians to suspect CES when one or more of the listed red flags are present and to refer these patients for further investigation. There are some limitations to this approach however which may lead to missed diagnoses. The ‘flags’ listed are often quite limited in description which may lead to limited scope of questioning and examination. For example, the inclusion of the specific term ‘saddle anaesthesia’ in red flag guidance, and its absence on a subsequent focused examination, may cause the patient with more subtle impaired perineal sensation, or alternative sensory loss in the lower limbs to go undetected, if its absence deters from a full dermatomal assessment being carried out. Some of the symptoms associated with CES, especially those regarding urinary and sexual dysfunction, are ones that patients may be uncomfortable describing organically, and as such, a list of generic screening questions may be a more favourable way of screening for symptoms, in place of listed ‘red flags’.

Furthermore, these red flag symptoms often describe the late features of CES or the features of CESR. These features, deemed ‘white flags’ by Todd [47], are of late irreversible disease with low prospect of recovery. As Todd theorised [47], if clinicians were to wait for these late symptoms, such as urinary incontinence, to appear before referring a patient, the patient cohort with the most potential gain from surgical intervention would be missed. Considering Markham [48] ‘window for opportunity for surgery’ as opening ‘at the onset of urinary or bowel dysfunction’, we would agree with Todd that the early symptoms, or ‘true red flags’, should instead be the focus of our screening for CES.

Alongside this, it is not often clear which, if any, of these symptoms may qualify as rule-in and rule-out symptoms as there is often no clinical weight or timing attributed to each. Some guidance, such as that from the BMJ [34], organises symptoms by frequency of presentation but most simply provide a list of potentially alarming clinical findings. As Barraclough describes, perhaps the ideal prescriptive advice would describe early symptoms of CES [49] or perhaps would even rationale the timing of symptoms by using Lavy’s extended subclasses of definition [43]. Further study may be required to assess which symptoms carry the most clinical weight at different stages of CES and therefore which should prompt increased alarm accordingly.

Interestingly whilst there was a consensus surrounding urinary dysfunction as a screening symptom of CES, only three of the sources elaborated on the investigation of this, specifically mentioning using post-void residual (PVR) scanning in the work-up of CES patients [29, 35, 39]. Two recent studies, by Todd et al. [50] and Venkatesan et al.  [51] respectively, have investigated the use of PVR values in predicting CES. Venkatesan et al.’s investigations found that amongst their patient cohort, a PVR < 200 mL had a negative predictive value of 97% for CES, which compared favourably to the negative predictive value of a clinical exam that assessed anal tone or perianal sensation. The authors would agree with Todd et al. that whilst further investigation of PVR as a prognostic tool is warranted, given the inaccuracy of clinical examination in diagnosis of CES [43], that the addition of early PVR scanning, in conjunction with traditional clinical examination, may be a valuable inclusion in any potential screening pathway.

As detailed in the introduction to this paper, the speed in which a diagnosis of CES can be made, and decompressive surgery commenced, is critical to ensuring favourable patient outcomes. From the results of our study, there is a clear consensus for MRI scanning of the spine as the gold standard immediate investigative action, with CT myelogram scanning as a second line if required. Whilst this unison in the choice of investigation is informative, it is concerning when we consider the poor access for clinicians to emergency MRI scanning in Ireland, particularly in out-of-hours scenarios. A 2011 review into the provision of appropriate spinal imaging [52] highlighted the paucity in availability of the emergency imaging modalities, finding that in the reviewed 34 national hospitals, only 5% (2/34) and 47% (16/34) had access to out-of-hours MRI scanning and CT scanning, respectively. Even in regular working hours, the access to MRI was seen to be relatively poor, with only 50% (17/34) of surveyed hospitals able to provide MRI services. Similarly, whilst several of the UK-based guidelines surveyed recommended 24-h access to MRI scanning [33, 35], a 2013 survey of UK hospitals found that only 14% were able to provide full out-of-hours access to MRI services. It is clear that a key rate-limiting step in this pathway is often the provision of emergency MRI, at potential expense to patient well-being.

The actual number of potential CES patients scanned who go onto receive urgent surgery is indeed relatively low, estimated at 13% by one UK-based review [53]. However, reasonably referred scans with negative findings and no subsequent intervention should not be viewed as a waste of provisions, in the situation where the scan is to rule out such a potentially catastrophic diagnosis. Systemising the referral pathway to help physicians be consistent as they rationalise which patients require investigation may have the bonus of avoiding over-referrals and preventing further logjam in the provision of MRI services. Whilst the authors note that costs regarding scanning and adequate staffing may be challenges in improving MRI provision, any analysis of the financial burden of providing improved availability to MRI services should incorporate the grave clinical and financial implications of missed CES, especially the legal cost of cases deemed inadequately investigated.

For a relatively rare condition, CES carries a large medicolegal and financial burden. The State Claims Agency of Ireland’s Clinical Risk Insights details 41 finalised claims in the 10-year period between January 2008 and December 2018 inclusive, with the paid amount totalling €20,901,261 (an average of €509,787 per claim) [25].

This is not a phenomenon unique to Ireland, with claims in the UK in a 3-year period between 2013 and 2016 estimated to have cost £68 million in total [54]. This legal toll is disproportionately large, with one UK-based review [54] showing that approximately 23% of NHS claims relating to spinal surgery were due to cases of CES, a condition with a relatively low incidence rate. Similarly, 65% of reports notified to the Medical Defence Union (MDU) in the UK resulted in a claim, with 48% of these resulting in payment, proportional outcomes much higher than the mean for all UK-based claims [48].

The national cost of litigation related to adverse medical outcomes is a clearly worsening problem in Ireland. In March of this year, it was widely reported by Irish media outlets, from numbers released by the State Claims Agency that the country’s liability for outstanding legal claims had totalled €4.957 billion, with €3.86 billion of this directly related to medical negligence cases. CES cases admittedly constitute a small minority of this total, but one that could be avoided with the institution of proper best practices. In an increasingly litigated field, the absence of a universal approach amongst physicians, by way of a standardised screening pathway, causes not only a clinical burden but a financial one for patients, clinicians and the state at large. Whilst the causes and details of all individual claims are not available, Markham [48] does describe that ‘virtually all of these (MDU) cases involved incorrect or delayed diagnosis’, and it is the authors opinion that the publication of new comprehensive screening pathway could help lessen delays and misdiagnoses and relieve the medicolegal burden.

We acknowledge that this review had some limitations. As our search was limited to guidance or advice that had been published or was available for translation in the English language, it was difficult to conduct a truly international review of what screening tools were available. It should be noted whilst the GIRFT template would be a fine template to follow for designing further guidance, it is possible that other more effective untranslated tools exist. In addition, the lack of access to case-specific legal information was a hindrance to truly analysing the effects of a lack of guidance in Ireland. Whilst overall state legal costs were available, details of individual cases were not, whether that be due to the nature of many medicolegal cases culminating in settlements, or the fact that such cases were currently active.

The recent publication in February 2023 of the UK’s NHS GIRFT CES pathway [35] is a promising development in the screening for CES for British- or UK-based clinicians. This, the most recently published guidance reviewed in this study, incorporates a stepwise format, detailing the standards of care from primary presentation through to post-operative care. In particular, the ‘click-through’ interactive manner of the pathway is innovative, with hyperlinks included to helpful resources, including MRI safety checks, a neurogenic bowel function guideline and support information and groups for CES patients. Bearing in mind the topic of litigation in the case of less favourable CES patient outcomes, the inclusion of a safety netting guide, alongside advice regarding consenting, is another welcome addition in the GIRFT screening pathway. It should also be noted also that previous updates to both the BASS/SBNS and NICE guidelines, after consultation with the Medical Protection Society (MPS), have lowered the bar for investigation by MRI, indicating an ongoing international trend towards more comprehensive screening, and a growing awareness of the potential damage caused by its absence.

## Conclusion

Overall, the authors feel we met the objectives that were set out at the onset of this project. We managed to collate and compare a broad range of guidelines and sources of guidance from several reputable international sources. We analysed the offered guidance and contrasted them with what information was available in recent and relevant literature. In addition, we believe this review has highlighted the dearth of support for clinicians in Ireland relating to the detection of CES and the need for further review and implementation of a structured clinical guidance.

On review, there is a notable lack of guidance for clinicians in screening for the symptoms of CES in Ireland. Whilst MRI scanner availability and access to surgical treatment may be time-limiting factors in treatment of CES, delays in diagnoses or misdiagnoses due to the absence of a suitable screening tool should be avoided. It is clear from our review that the fallout, whether it be patient morbidity, legal procession, or financial loss, from missed or misdiagnosis of CES cases is enormous and with proper standardised precautions, could be lessened.

The recent publication of the GIRFT pathway in the UK is a promising development and one that we should consider when formulating further guidance. Our recommendation is that any guidance drafted should incorporate a similar step-through authorised pathway, with prescriptive advice focusing on early symptoms of CES, advice towards clinical examination, consistent documentation and a low threshold for emergency MRI scanning. Where urgent and out-of-hours MRI scanning is not available, contingency standard operating procedures should be in place for immediate transfer for emergency scanning. Similar procedural planning should be in place to cater for prompt surgical intervention where warranted.

In conclusion, it is the authors’ opinion that national consensus on thorough screening and prompt investigation for CES is necessary, and the formulation of a comprehensive new CES screening pathway for clinicians in Ireland would be a welcome addition to what is currently available and would enhance the overall quality of healthcare surrounding this condition.

## References

[1] Podnar S (2007) Epidemiology of cauda equina and conus medullaris lesions. Muscle Nerve 35(4):529–31. 10.1002/mus.20696 17143890 10.1002/mus.20696

[2] Ahn UM, Ahn NU, Buchowski JM et al (2000) Cauda equina syndrome secondary to lumbar disc herniation. Spine 25(12):1515–1522. 10.1097/00007632-200006150-00010 10851100 10.1097/00007632-200006150-00010

[3] Kostuik JP (2004) Medicolegal consequences of cauda equina syndrome: an overview. Neurosurgical Focus FOC 16(6):39–41. 10.3171/foc.2004.16.6.7 10.3171/foc.2004.16.6.7 15202878

[4] Malmivaara A, Pohjola R (1982) Cauda equina syndrome caused by chiropraxis on a patient previously free of lumbar spine symptoms. Lancet 2(8305):986–987. 10.1016/s0140-6736(82)90184-2 6127483 10.1016/s0140-6736(82)90184-2

[5] Harrop JS, Hunt GE, Vaccaro AR (2004) Conus medullaris and cauda equina syndrome as a result of traumatic injuries: management principles. Neurosurg Focus 16(6):1–23 10.3171/foc.2004.16.6.4 15202874

[6] Jensen RL (2004) Cauda equina syndrome as a postoperative complication of lumbar spine surgery. Neurosurgical Focus FOC 16(6):34–38. 10.3171/foc.2004.16.6.6 10.3171/foc.2004.16.6.6 15202877

[7] Özgen S, Baykan N, Dogan IV et al (2004) Cauda equina syndrome after induction of spinal anesthesia. Neurosurgical Focus FOC 16(6):1–27. 10.3171/foc.2004.16.6.5 10.3171/foc.2004.16.6.5 15202875

[8] Lenehan B, Sullivan P, Street J, Dudeney S (2005) Epidural abscess causing cauda equina syndrome. Ir J Med Sci 174(3):88–91. 10.1007/bf03169156 16285347 10.1007/bf03169156

[9] Cohen DB (2004) Infectious origins of cauda equina syndrome. Neurosurg Focus 16(6):e2. 10.3171/foc.2004.16.6.2 15202872 10.3171/foc.2004.16.6.2

[10] Wright MH, Denney LC (2003) A comprehensive review of spinal arachnoiditis. Orthop Nurs 22(3):215–9; quiz 20–1. 10.1097/00006416-200305000-00010 12803151 10.1097/00006416-200305000-00010

[11] Kebaish KM, Awad JN (2004) Spinal epidural hematoma causing acute cauda equina syndrome. Neurosurg Focus 16(6):e1 15202871

[12] Belinchón JM, Campos J, Merino J et al (2005) Chronic spontaneous lumbar epidural hematoma. Neurocirugia (Astur) 16(6):533–536 16378137 10.1016/S1130-1473(05)70384-5

[13] McCabe FJ, Jadaan DY, Jadaan MM, McCabe JP (2020) The rise of metastatic bone disease in Ireland. Clin Exp Metastasis 37(6):693–702. 10.1007/s10585-020-10059-7 33099723 10.1007/s10585-020-10059-7

[14] Fraser S, Roberts L, Murphy E (2009) Cauda equina syndrome: a literature review of its definition and clinical presentation. Arch Phys Med Rehabil 90(11):1964–1968. 10.1016/j.apmr.2009.03.021 19887225 10.1016/j.apmr.2009.03.021

[15] Hoeritzauer I, Wood M, Copley PC et al (2020) What is the incidence of cauda equina syndrome? A systematic review. J Neurosurg Spine 32(6):832–841. 10.3171/2019.12.Spine19839 32059184 10.3171/2019.12.Spine19839

[16] Woodfield J, Lammy S, Jamjoom AAB et al (2022) Demographics of cauda equina syndrome: a population-based incidence study. Neuroepidemiology 56(6):460–468. 10.1159/000527727 36315989 10.1159/000527727 PMC9945186

[17] Gardner A, Gardner E, Morley T (2011) Cauda equina syndrome: a review of the current clinical and medico-legal position. Eur Spine J 20(5):690–697. 10.1007/s00586-010-1668-3 21193933 10.1007/s00586-010-1668-3 PMC3082683

[18] Kohles SS, Kohles DA, Karp AP et al (2004) Time-dependent surgical outcomes following cauda equina syndrome diagnosis: Comments on a meta-analysis. Spine (Phila Pa 1976) 29(11):1281–7. 10.1097/00007632-200406010-00019 15167669 10.1097/00007632-200406010-00019

[19] Shapiro S (2000) Medical realities of cauda equina syndrome secondary to lumbar disc herniation. Spine (Phila Pa 1976) 25(3):348–51; discussion 52. 10.1097/00007632-200002010-00015 10703108 10.1097/00007632-200002010-00015

[20] Olivero WC, Wang H, Hanigan WC et al (2009) Cauda equina syndrome (CES) from lumbar disc herniations. J Spinal Disord Tech 22(3):202–206. 10.1097/BSD.0b013e31817baad8 19412023 10.1097/BSD.0b013e31817baad8

[21] Dinning TA, Schaeffer HR (1993) Discogenic compression of the cauda equina: a surgical emergency. Aust N Z J Surg 63(12):927–934. 10.1111/j.1445-2197.1993.tb01721.x 8285904 10.1111/j.1445-2197.1993.tb01721.x

[22] Buchner M, Schiltenwolf M (2002) Cauda equina syndrome caused by intervertebral lumbar disk prolapse: mid-term results of 22 patients and literature review. Orthopedics 25(7):727–731. 10.3928/0147-7447-20020701-12 12138958 10.3928/0147-7447-20020701-12

[23] Fairbank J, Hashimoto R, Dailey A et al (2011) Does patient history and physical examination predict MRI proven cauda equina syndrome? Evid Based Spine Care J 2(4):27–33. 10.1055/s-0031-1274754 23230403 10.1055/s-0031-1274754 PMC3506147

[24] Boden SD, Davis DO, Dina TS et al (1990) Abnormal magnetic-resonance scans of the lumbar spine in asymptomatic subjects. A prospective investigation. J Bone Joint Surg Am 72(3):403–8 2312537 10.2106/00004623-199072030-00013

[25] McCullagh MOKC, Synnott K, Grady C et al (2020) Cauda equina syndrome. Clinical Risk Insights. State Claims Agency [Internet Article], 2nd edn. Winter pp 4–5. https://stateclaims.ie/uploads/banner/State-Claims-Agency-Clinical-Risk-Insights_Winter-2020.pdf. Accessed 14 Mar 2023

[26] Excellence NIfHCa. Sciatica (lumbar radiculopathy) red flag symptoms and signs [Web Published Guideline]. http://cks.nice.org.uk/sciatica-lumbar-radiculopathy. National Institute for Health Care and Excellence 2022 [updated February 2022. NICE Guideline]. Accessed 14 Mar 2023

[27] Longmore M, Wilkinson I, Davidson E et al (2010) Oxford Handbook of Clinical Medicine, 8th edn. Oxford University Press

[28] Staten A, Staten P (2020) Practical general practice - Guidelines for effective clinical management, 7th edn. Elsevier

[29] Bridwell KH, DeWald RL (1992) The textbook of spinal surgery. J Pediatr Orthop 12(4):550. 10.1097/01241398-199207000-00035 10.1097/01241398-199207000-00035

[30] Frymoyer JW, Lauerman WC, Wiesel SW et al (2023) Adult and pediatric spine. Wolters Kluwer Health, Philadelphia, USA

[31] Hsu PSAC, Levin K (2023) Acute lumbosacral radiculopathy: pathophysiology, clinical features, and diagnosis. In: Post TE (ed). UpToDate Inc., Waltham, MA, USA

[32] Levin K, Hsu PS, Armon C (2023) Acute lumbosacral radiculopathy: treatment and prognosis. In: Post TE (ed). UpToDate Inc., Waltham, MA, USA

[33] Society of British Neurological Surgeons BAoSS (2018) Standards of Care for Investigation and Management of Cauda Equina Syndrome [Online Guideline]. https://spinesurgeons.ac.uk/resources/Documents/News/Cauda_Equina_Syndrome_Standards_SBNS_BASS%20-%20Dec%202018.pdf. Society of British Neurological Surgeons, British Association of Spine Surgeons [updated 2018]. Accessed 14 Mar 2023

[34] Cassey KF (2023) Cauda Equina Syndrome. Brit Med J. https://bestpractice.bmj.com/topics/en-us/3000164. Accessed 14 Mar 2023

[35] National Health Service UK. National Suspected Cauda Equina Syndrome Pathway. https://gettingitrightfirsttime.co.uk/. Accessed 14 Mar 2023

[36] North Devon District Hospital (Department of Trauma and Orthopaedics) (2020) Guidelines for the management of suspected cauda equina syndrome & decompensating spinal stenosis at NDDH. NHS North Devon; 2023. https://www.northdevonhealth.nhs.uk/wp-content/uploads/2020/02/GUIDEL1.pdf. (Updated 2020 Feb; 2.1 [Local Guideline]). Accessed 14 Mar 2023

[37] Hutchins TA, Peckham M, Shah LM et al (2021) ACR Appropriateness Criteria^®^ Low Back Pain: 2021 Update. J Am Coll Radiol 18(11 Supplement):S361–S79. 10.1016/j.jacr.2021.08.002 34794594 10.1016/j.jacr.2021.08.002

[38] Wiseman D (2023) Cauda Equina Syndrome. American Association of Neurological Surgeons. https://www.aans.org/Patients/Neurosurgical-Conditions-and-Treatments/Cauda-Equina-Syndrome. Accessed 14 Mar 2023

[39] Forsthoefel C, Moore DW (2023) Cauda Equina Syndrome www.orthobullets.com. Available from: https://www.orthobullets.com/spine/2065/cauda-equina-syndrome. (Updated 26 Apr 2023). Accessed 14 Mar 2023

[40] Tandon P, Sankaran B (1967) Cauda equina syndrome due to lumbar disc prolapse. Indian J Orthop 1(02):112–119

[41] Shi J, Jia L, Yuan W et al (2010) Clinical classification of cauda equina syndrome for proper treatment. Acta Orthop 81(3):391–5. 10.3109/17453674.2010.483985 20443745 10.3109/17453674.2010.483985 PMC2876846

[42] Gleave JRW, MacFarlane R (2002) Cauda equina syndrome: what is the relationship between timing of surgery and outcome? Br J Neurosurg 16(4):325–328. 10.1080/0268869021000032887 12389883 10.1080/0268869021000032887

[43] Lavy C, Marks P, Dangas K, Todd N (2022) Cauda equina syndrome-a practical guide to definition and classification. Int Orthop 46(2):165–169. 10.1007/s00264-021-05273-1 34862914 10.1007/s00264-021-05273-1 PMC8782783

[44] Ahad A, Elsayed M, Tohid H (2015) The accuracy of clinical symptoms in detecting cauda equina syndrome in patients undergoing acute MRI of the spine. Neuroradiol J 28(4):438–442. 10.1177/1971400915598074 26306934 10.1177/1971400915598074 PMC4757302

[45] Bell DA, Collie D, Statham PF (2007) Cauda equina syndrome: what is the correlation between clinical assessment and MRI scanning? Br J Neurosurg 21(2):201–203. 10.1080/02688690701317144 17453789 10.1080/02688690701317144

[46] Gooding BW, Higgins MA, Calthorpe DA (2013) Does rectal examination have any value in the clinical diagnosis of cauda equina syndrome? Br J Neurosurg 27(2):156–9. 10.3109/02688697.2012.732715 23113877 10.3109/02688697.2012.732715

[47] Todd NV (2017) Guidelines for cauda equina syndrome. Red flags and white flags. Systematic review and implications for triage. Br J Neurosurg 31(3):336–9. 10.1080/02688697.2017.1297364 28637110 10.1080/02688697.2017.1297364

[48] Markham DE (2004) Cauda equina syndrome: Diagnosis, delay and litigation risk. Curr Orthop 18(1):58–62. 10.1016/j.cuor.2003.10.006 10.1016/j.cuor.2003.10.006

[49] Barraclough K (2021) Cauda equina syndrome. BMJ 372:n32. 10.1136/bmj.n32 33436390 10.1136/bmj.n32

[50] Todd N, Dangas K, Lavy C (2022) Post-void bladder ultrasound in suspected cauda equina syndrome—Data from medicolegal cases and relevance to magnetic resonance imaging scanning. Int Orthop 46(6):1375–1380. 10.1007/s00264-022-05341-0 35182176 10.1007/s00264-022-05341-0 PMC9117366

[51] Venkatesan M, Nasto L, Tsegaye M, Grevitt M (2019) Bladder scans and postvoid residual volume measurement improve diagnostic accuracy of cauda equina syndrome. Spine. 44(18):1303–8 31479434 10.1097/BRS.0000000000003152

[52] Kelly JC, O’Briain DE, Kelly GA, Mc Cabe JP (2012) Imaging the spine for tumour and trauma – a national audit of practice in Irish hospitals. Surgeon 10(2):80–3. 10.1016/j.surge.2011.01.007 22385529 10.1016/j.surge.2011.01.007

[53] Hussain MM, Razak AA, Hassan SS et al (2018) Time to implement a national referral pathway for suspected cauda equina syndrome: Review and outcome of 250 referrals. Brit J Neurosurg. 32(3):264–8. 10.1080/02688697.2018.1457771 29607679 10.1080/02688697.2018.1457771

[54] Machin JT, Hardman J, Harrison W et al (2018) Can spinal surgery in England be saved from litigation: A review of 978 clinical negligence claims against the NHS. Eur Spine J 27(11):2693–2699. 10.1007/s00586-018-5739-1 30151803 10.1007/s00586-018-5739-1

